# Association of Mutations Contributing to Clonal Hematopoiesis With Prognosis in Chronic Ischemic Heart Failure

**DOI:** 10.1001/jamacardio.2018.3965

**Published:** 2018-12-19

**Authors:** Lena Dorsheimer, Birgit Assmus, Tina Rasper, Christina A. Ortmann, Andreas Ecke, Khalil Abou-El-Ardat, Tobias Schmid, Bernhard Brüne, Sebastian Wagner, Hubert Serve, Jedrzej Hoffmann, Florian Seeger, Stefanie Dimmeler, Andreas M. Zeiher, Michael A. Rieger

**Affiliations:** 1 Department of Medicine, Hematology/Oncology, Goethe University Hospital, Frankfurt, Germany; 2 Department of Medicine, Cardiology, Goethe University Hospital, Frankfurt, Germany; 3 German Center for Cardiovascular Research, Berlin (partner site Frankfurt Rhine-Main), Germany; 4 Institute for Cardiovascular Regeneration, Goethe University, Frankfurt, Germany; 5 German Cancer Consortium, German Cancer Research Center, Heidelberg, Germany; 6 Institute for Biochemistry I, Goethe University Hospital, Frankfurt, Germany

## Abstract

**Importance:**

Somatic mutations causing clonal expansion of hematopoietic cells (clonal hematopoiesis of indeterminate potential [CHIP]) are increased with age and associated with atherosclerosis and inflammation. Age and inflammation are the major risk factors for heart failure, yet the association of CHIP with heart failure in humans is unknown.

**Objective:**

To assess the potential prognostic significance of CHIP in patients with chronic heart failure (CHF) owing to ischemic origin.

**Design, Setting, and Participants:**

We analyzed bone marrow–derived mononuclear cells from 200 patients with CHF by deep targeted amplicon sequencing to detect the presence of CHIP and associated such with long-term prognosis in patients with CHF at University Hospital Frankfurt, Frankfurt, Germany. Data were analyzed between October 2017 and April 2018.

**Results:**

Median age of the patients was 65 years. Forty-seven mutations with a variant allele fraction (VAF) of at least 0.02 were found in 38 of 200 patients with CHF (18.5%). The somatic mutations most commonly occurred in the genes *DNMT3A* (14 patients), *TET2* (9 patients), *KDM6A* (4 patients), and *BCOR* (3 patients). Patients with CHIP were older and more frequently had a history of hypertension. During a median follow-up of 4.4 years, a total of 53 patients died, and 23 patients required hospitalization for heart failure. There was a significantly worse long-term clinical outcome for patients with either *DNMT3A* or *TET2* mutations compared with non-CHIP carriers. By multivariable Cox proportional regression analysis, the presence of somatic mutations within *TET2* or *DNMT3A* (HR, 2.1; 95% CI, 1.1-4.0; *P* = .02, for death combined with heart failure hospitalization) and age (HR, 1.04; 95% CI, 1.01-1.07 per year; *P* = .005) but not a history of hypertension remained independently associated with adverse outcome. Importantly, there was a significant dose-response association between VAF and clinical outcome.

**Conclusions and Relevance:**

Our data suggest that somatic mutations in hematopoietic cells, specifically in the most commonly mutated CHIP driver genes *TET2* and *DNMT3A*, may be significantly associated with the progression and poor prognosis of CHF. Future studies will have to validate our findings in larger cohorts and address whether targeting specific inflammatory pathways may be valuable for precision medicine in patients with CHF carrying specific mutations encoding for CHIP.

## Key Points

**Question:**

What is the clinical significance of clonal hematopoiesis of indeterminate potential (CHIP) for chronic heart failure (CHF) owing to ischemic origin?

**Findings:**

In this cohort study, CHIP had a high prevalence in 200 investigated patients with CHF. While no clinical baseline characteristics associated with CHF were different between CHIP carriers and non-CHIP carriers, except for the mean age, harboring mutations in the most prevalent driver genes associated with CHIP, namely *DNMT3A* and *TET2*, was associated with a significant and profound increase in death and rehospitalization for heart failure.

**Meaning:**

Clonal hematopoiesis of indeterminate potential is presented as a newly identified risk factor for impaired long-term survival and increased disease progression in patients with CHF that may be well targetable as a valuable approach to precision medicine in patients with CHF carrying specific mutations encoding for clonal hematopoiesis.

## Teaser

This study assesses the potential prognostic significance of clonal hematopoiesis of indeterminate potential in patients with chronic heart failure owing to ischemic origin.

## Introduction

Clonal hematopoiesis of indeterminate potential (CHIP) is defined as the presence of an expanded somatic blood cell clone in persons without other hematological abnormalities.^1,2,3^ The presence of CHIP was shown to increase with age and is significantly associated with the risk for coronary heart disease.^2,4^ The most commonly mutated genes in CHIP are the DNA methyltransferase *DNMT3A* and the DNA demethylase *TET2*, which both epigenetically control gene expression. Functionally, these 2 predominant CHIP driver genes are important regulators of inflammation by augmenting monocyte adhesion and stimulated macrophage activity.^5,6^ Accordingly, loss of *TET2* function in hematopoietic cells accelerates atherosclerosis and promotes inflammasome activation in mice,^4,7^ whereas loss of *DNMT3A* experimentally altered myeloid cell function and promoted upregulation of chemokines.^8,9^

Aging is the major risk factor for heart failure,^10^ and inflammation importantly contributes to the progression of ischemic heart failure.^11^ However, to our knowledge, there are no data assessing the incidence and potential prognostic significance of the presence of CHIP in patients with chronic heart failure (CHF) owing to ischemic origin. Therefore, we used targeted amplicon sequencing to detect the presence of CHIP in bone marrow–derived mononuclear cells (BMC) and associated such presence with long-term prognosis in patients with chronic ischemic heart failure.

## Methods

### Study Cohort

Clinical data and biological specimens (BMC) were collected from a total of 200 patients with CHF and participating in different trials examining the effects of intracoronary administration of autologous BMCs between June 2005 and July 2017 at the University Hospital of the Goethe University, Frankfurt/Main, Germany. All patients provided written informed consent for 1 of the following registered clinical trials: Transplantation of Progenitor Cells and Recovery of Left Ventricular Function in Patients with Chronic Ischemic Heart Disease (TOPCARE-CHD; Crossover or Registry; n = 134; NCT00289822 or NCT00962364^12,13^), Cellwave (n = 55; NCT00326989^14^) or Repetitive Progenitor Cell Therapy in Advanced Chronic Heart Failure (REPEAT; n = 11; NCT01693042^13^). The selection of patients from the individual parent trials is illustrated in the eFigure in the Supplement. In addition to the clinical trials, patients provided additional informed consent for genetic testing of bone marrow samples. The ethics review board of the Goethe University in Frankfurt, Germany, approved the protocols. The study complies with the Declaration of Helsinki. Patients were eligible for inclusion into the study if they had stable CHF symptoms New York Heart Association (NYHA) classification of at least II, had a previous successfully revascularized myocardial infarction at least 3 months before bone marrow aspiration, and had a well-demarcated region of left ventricular dysfunction on echocardiography. Exclusion criteria were the presence of acutely decompensated heart failure with NYHA class IV, an acute ischemic event within 3 months prior to inclusion, a history of severe chronic diseases, documented cancer within the preceding 5 years, or unwillingness to participate.

Clinical data, medication, and laboratory data were prospectively collected. Follow-up visits were scheduled at 3 to 4 months after cell application and at 12 months after cell application and were performed by physicians, whereas follow-up telephone calls were performed by study nurses at 18, 24, 36, and 48 months. The Seattle Heart Failure Model (SHFM) score was calculated by including age, sex, etiology of cardiomyopathy (ischemic origin), heart rate, systolic blood pressure, ejection fraction, medication (angiotensin-converting enzyme inhibitor, angiotensin receptor blocker, aldosterone blocker, β-blocker, statins, diuretic type and daily dose, and allopurinol), and laboratory values (serum sodium, total cholesterol, hemoglobin, percent lymphocytes, and uric acid). In addition, the presence of any implantable device (pacemaker, implantable cardioverter-defibrillator, or cardiac resynchronization therapy) is included into the calculation of the SHFM Score. N-terminal pro b-type natriuretic peptide (NT-proBNP) serum levels were measured at the time of bone marrow harvest.

Mortality and mode of death were adjudicated by means of reviewing medical records by the study physicians. Mode of death was classified as sudden death (unexpected death in a clinically stable patient, typically within 1 hour of symptom onset, from documented or presumed cardiac arrhythmia and without a clear noncardiovascular cause), pump failure (progressively reduced cardiac output and failure of organ perfusion), noncardiac death, or not classifiable. Missing data on survival were obtained by contacting the federal registration offices. Rehospitalization for heart failure was defined as an at least 24 hours’ hospital stay for heart failure symptoms with documented change in heart failure–directed medication. Physicians assessing heart failure admissions were unaware of the mutation status of the patients.

### Sample Preparation for Next-Generation Sequencing

Bone marrow aspirate (50 mL) was obtained from the iliac crest with local anesthesia. Bone marrow–derived mononuclear cells were isolated by Ficoll density-gradient centrifugation, as previously reported.^12,15^

DNA was isolated with QIAamp DNA Mini Kit (Qiagen) from deep-frozen samples of BMC and peripheral blood. The concentration of the extracted genomic DNA was determined using the Qubit dsDNA HS Assay Kit (Life Technologies) on a Qubit Fluorometer (Life Technologies).

A custom panel based on the Illumina TruSeq Custom Amplicon Low Input assay was designed to assess the presence of CHIP in the patients. The panel includes 594 amplicons in 56 genes (eTable 1 in the Supplement) commonly mutated in CHIP and myeloid malignancies. To allow improved identification of low allele frequency variants, a dual-strand approach was used. In addition, 6-bp unique molecular identifiers (UMIs) were included in the target specific primers.

Target enrichment was performed from 40-ng genomic DNA according to the instructions of the TruSeq Custom Amplicon Low Input Kit (Illumina). The hybridization of the oligo pool to the target regions, the extension from the upstream oligo, and the following ligation to the 5′ end of the downstream oligo was performed on a T100 Thermal Cycler (Bio-Rad Laboratories). Amplification of the ligation products and addition of the Illumina i7 and i5 adapter sequences was performed on a T100 Thermal Cycler (Bio-Rad Laboratories). After purification, library quality and size distribution were assessed on a Bioanalyzer 2100 (Agilent Technologies) using the DNA 1000 Kit. Bead-based library normalization was performed before pooling of the individual libraries.

### High-Throughput Sequencing

Before sequencing, the pooled libraries were diluted and denatured according to the NextSeq System Denature and Dilute Libraries Guide (Illumina) and 1% PhiX DNA was added. The pooled libraries were sequenced on a NextSeq 500 sequencer (Illumina) using the NextSeq 500/550 Mid Output, version 2 kit (300 cycles) according to the manufacturer’s instructions. The sequencer was operated in a paired-end sequencing mode with 2 × 150 bp read length and 2 × 8 bp index read length. The BCL files were demultiplexed and converted to FASTQ files using the FASTQ Generation tool on BaseSpace (Illumina). The median coverage across all BMC samples was 4282× before UMI family clustering and 630× with inclusion of UMIs.

### Variant Calling and Annotation Strategies

Read quality was assessed using FastQC.^16^ FASTQ files from each patient were merged and reads were grouped into unique molecular identifier (UMI) families using the UMI-tools software package.^17^ Reads were mapped to the hg19 draft of the human genome using Burrows-Wheeler Alignment–MEM. The `dedup` command of the UMI-tools software package was used to remove polymerase chain reaction duplicates with the same UMIs and alignment coordinates. Variant calling was performed using FreeBayes^18^ without allele frequency threshold, a minimum alternate read count of 2, and a minimum base and mapping quality of 20. Variant effect prediction and variant annotation was performed using SnpEff and SnpSift.^19^

The identified variants were processed and filtered using the R programming language, version 3.3.1 (R Programming). Common single-nucleotide polymorphisms with a minor allele frequency of at least 5% in either the 1000 Genome Project, Exome Variant Server, or ExAC databases were excluded. In addition, variants with a low mapping quality (<20) and variants occurring in 8% or more of the patients in the studied cohort were considered as technical artifacts and excluded. Furthermore, variants covered with fewer than 100 reads in at least 1 set of amplicons (CAT A or CAT B), variants called with only 1 of the set of amplicons (CAT A or CAT B), single-nucleotide polymorphisms identified as common in the single-nucleotide polymorphism database (≥1% in the human population), and variants with sequence ontology terms “LOW” or “MODIFIER” were filtered out. According to previously established definitions,^3^ all variants with a variant allele fraction (VAF) of at least 0.02 (2%) were considered; VAF was calculated by using the formula VAF = alternate reads / (reference + alternate reads). Variants with a VAF of 0.45 to 0.55 were not considered to exclude potential germline variants.

The variants were further validated on the basis of being reported in the literature and/or the Catalogue of Somatic Mutations in Cancer (https://cancer.sanger.ac.uk/cosmic) and ClinVar (https://www.ncbi.nlm.nih.gov/clinvar).

### Statistical Analysis

Continuous variables are presented as mean (SD), and as median (interquartile range [IQR]) unless otherwise noted. Analysis of variance testing was used for comparison of continuous variables between groups. Categorical variables were compared with the χ^2^ test or Fisher exact test as appropriate.

For survival analysis within the individual groups, Kaplan-Meier analyses were used. Log rank testing was applied for comparison of event-free survival analysis. In addition, a multivariable Cox proportional-hazards regression model was used to calculate hazard ratios (HRs) for event rates, using the univariate significant factors associated with adverse outcome, namely age and a history of hypertension, as covariates. In addition, to comprehensively adjust for potentially confounding prognostic factors in CHF, a multivariate stepwise Cox regression analysis was performed including baseline serum NT-proBNP levels (stepwise approach using steps of 1000 pg/mL [to convert to nanograms per liter, multiply by 1]) as well as the SHFM score tertiles. Statistical significance was assumed at *P* less than .05, and all reported *P* values are 2-sided. Statistical analysis was performed with SPSS, version 23.0 (SPSS Inc).

## Results

### Patient Characteristics and Detection of Mutations

To examine whether CHIP plays a potential role in CHF, we analyzed BMCs from 200 patients with CHF by targeted amplicon sequencing of 56 genes associated with CHIP and myeloid malignancies (eTable 1 in the Supplement). Patients had a median age of 65 years, NYHA class II, and a left ventricular ejection fraction of 31% owing to a previous myocardial infarction (median time since last infarction, 5 years; Table 1). Median time of follow-up was 4.4 years (IQR, 2.8-5).

**Table 1.** Baseline Characteristics

Characteristic^a^	Total Cohort (n = 200)	CHIP (n = 38)	Non-CHIP (n = 162)	*P* Value (CHIP vs Non-CHIP)
Age, y (n = 200)				
Mean (SD)	64 (10.7)	68.7 (8.2)	62.5 (10.8)	.001
Median (IQR)	65 (56 to 72)	69 (64 to 75)	63 (56 to 71)	
Sex, No. (n = 200)				
Male	169	32	137	.97
Female	31	6	25	
Hypertension, No. (%) (n = 199)	159 (80)	36 (95)	123 (76)	.01
Hyperlipidemia, No. (%) (n = 199)	161 (81)	32 (84)	129 (80)	.56
Diabetes mellitus, No. (%) (n = 199)	71 (36)	14 (37)	57 (35)	.87
Current or former smoking, No. (%) (n = 198)	129 (65)	26 (68)	103 (64)	.87
Family history of coronary artery disease, No. (%) (n = 197)	104 (53)	17 (45)	87 (55)	.27
Extent of coronary artery disease, No. (n = 197)				
1 vessel disease	50	5	45	.15
2 vessel disease	46	11	35	
3 vessel disease	101	22	79	
Weight, kg (n = 199)				
Mean (SD)	82.0 (14.5)	82.0 (16.1)	82.1 (14.1)	.97
Median (IQR)	81 (72 to 90)	82 (72 to 93)	80 (72 to 90)	
Size, cm (n = 199)				
Mean (SD)	173 (8)	171 (8)	173.9 (8)	.07
Median (IQR)	174 (168 to 178)	173 (167 to 177)	174 (169 to 179)	
Systolic blood pressure, mm Hg (n = 186)				
Mean (SD)	115 (23.0)	121.7 (25.8)	113.8 (22.1)	.06
Median (IQR)	112 (100 to 130)	117 (105 to 135)	111 (100 to 127)	
Diastolic blood pressure, mm Hg (n = 184)				
Mean (SD)	61.0 (10.5)	61.8 (10.2)	60.8 (10.6)	.64
Median (IQR)	60 (52 to 68)	61 (51 to 71)	60 (53 to 68)	
Heart rate, bpm (n = 171)				
Mean (SD)	67 (12)	65 (15)	67 (12)	.35
Median (IQR)	67 (60 to 72)	62 (59 to 75)	67 (60 to 72)	
NYHA class (n = 199)				
Mean (SD)	2.3 (0.7)	2.3 (0.7)	2.3 (0.7)	.84
Median (IQR)	2 (2 to 3)	2 (2 to 3)	2 (2 to 3)	
Time since last myocardial infarction, mo (n = 186)				
Mean (SD)	93 (90)	113 (98)	89 (87)	.16
Median (IQR)	65 (15 to 150)	100 (15 to 168)	60 (15 to 140)	
Seattle Heart Failure score (n = 181)				
Mean (SD)	0.4 (1.1)	0.4 (0.9)	0.4 (1.1)	.94
Median (IQR)	0.2 (−0.2 to 0.9)	0.2 (−0.1 to 1.0)	0.3 (−0.3 to 0.9)	
Left ventricular ejection fraction, % (n = 196)				
Mean (SD)	31.2 (11.2)	32.7 (12.2)	30.9 (11)	.38
Median (IQR)	30 (20 to 40)	32 (20 to 42)	30 (22 to 40)	
Severity of mitral regurgitation, grade (n = 171)				
Mean (SD)	0.8 (1)	0.8 (0.8)	0.8 (1)	.61
Median (IQR)	0.5 (0-1)	0 (0-1)	1.0 (0-1)	
NT-proBNP serum levels, pg/mL (n = 161)				
Mean (SD)	1971 (3559)	1714 (1741)	2032 (3872)	.64
Median (IQR)	953 (404 to 2019)	1010 (404 to 2875)	951 (404 to 1878)	
Creatinine, mg/dL (n = 188)				
Mean (SD)	1.3 (0.7)	1.2 (0.4)	1.3 (0.7)	.53
Median (IQR)	1.1 (1.0 to 1.4)	1.1 (1.0 to 1.4)	1.1 (1.0 to 1.4)	
High-sensitivity C-reactive protein levels, mg/dL (n = 197)				
Mean (SD)	0.68 (1.23)	0.73 (1.22)	0.66 (1.23)	.75
Median (IQR)	0.28 (0.12 to 0.68)	0.30 (0.15 to 0.67)	0.26 (0.12 to 0.68)	
High-sensitive troponin T levels, pg/mL (n = 65)				
Mean (SD)	14 (11)	17 (17)	14 (9)	.35
Median (IQR)	13 (5 to 20)	12 (3 to 27)	13 (6 to 20)	
Hemoglobin, g/dL (n = 132)				
Mean (SD)	14 (2)	13 (1)	14 (2)	.05
Median (IQR)	14 (13 to 15)	14 (12 to 14)	14 (14 to 15)	
Hematocrit, % (n = 132)				
Mean (SD)	42 (4)	40 (4)	42 (4)	.05
Median (IQR)	42 (39 to 45)	41 (37 to 43)	42 (39 to 45)	
Thrombocytes, /μL (n = 132)				
Mean (SD)	205 (63)	199 (52)	206 (65)	.60
Median (IQR)	203 (160 to 247)	190 (158 to 243)	206 (161 to 249)	
Leukocytes, /μL (n = 132)				
Mean (SD)	7.7 (2.4)	7.1 (1.6)	7.8 (2.6)	.23
Median (IQR)	7.4 (6.2 to 8.7)	7.1 (5.8 to 8.4)	7.4 (6.3 to 8.8)	

Abbreviations: CHIP, clonal hematopoiesis of indeterminate potential; IQR, interquartile range; NT-proBNP, N-terminal pro b-type natriuretic peptide.

SI conversion factors: To convert creatinine to micromoles per liter, multiply by 76.25; C-reactive protein to nanomoles per liter, multiply by 9.524; hematocrit to proportion of 1.0, multiply by 0.01; hemoglobin to grams per liter, multiply by 10; leukocytes to ×10^9 ^per liter, multiply by 0.001; NT-proBNP to nanograms per liter, multiply by 1; thrombocytes to ×10^9 ^per liter, multiply by 1.

^a^ Continuous variables are shown as mean (SD) and median (interquartile range). Categorical variables are shown as frequency (%).

**Figure 1.**
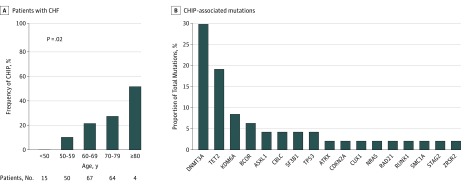
Prevalence of Clonal Hematopoiesis of Indeterminate Potential (CHIP) According to Age and Proportion of Mutated Genes Leading to Clonal Hematopoiesis in Patients With Chronic Ischemic Heart Failure (CHF) A, The number of patients with CHF analyzed per age group is given. *P* value is for analysis of variance test. B, CHIP-associated mutations in 56 analyzed genes that resulted in a variant allele fraction higher than 0.02 and remained positive after stringent unsupervised and supervised variant calling criteria are included. Because some patients carried more than 1 mutation, the total number of identified mutations leading to clonal expansion exceeds the number of patients with CHIP. The list of individual mutations can be found in eTable 3 in the Supplement.

Thirty-eight of 200 patients with CHF (18.5%) were CHIP carriers with a VAF of at least 0.02. Consistent with previous findings in the general population,^2^ the prevalence of CHIP in the present cohort of patients with CHF of ischemic origin increased with patient age (Figure 1A). However, compared with published cohorts of unselected populations^2^ as well as patients with coronary heart disease,^4^ the frequency of identified mutations was considerably higher in all age groups, with 10% of patients between age 50 and 59 years (5 of 50), 20.9% between age 60 and 69 years (14 of 67 patients), and 26.6% between age 70 and 79 years (17 of 64 patients) harboring CHIP-related mutations (Figure 1A). Thus, CHIP was enriched in patients with CHF of ischemic origin.

Forty-seven different somatic mutations with a VAF of at least 0.02 were identified (eTable 2 in the Supplement). The somatic mutations most commonly occurred in the genes *DNMT3A* (14 patients), *TET2* (9 patients), *KDM6A* (4 patients), and *BCOR* (3 patients) followed by *ASXL*, *CBLC*, *SF3B1*, and *TP53* (2 patients each). Nine other genes associated with CHIP were found to be mutated in individual patients (Figure 1B). Thirty of 38 participants (79%) with CHIP had a mutation in only a single driver gene, whereas 2 mutations were identified in 7 patients, and 1 patient even exhibited 3 mutations. The mean VAF was 0.065 (range, 0.02-0.23), which corresponds to 13.0% of nucleated blood cells harboring the mutation at the time of analysis, assuming all are heterozygous mutations.

In patients with *DNMT3A* and *TET2* mutations in their BMCs, we also verified the presence of the respective CHIP driver gene mutation in samples of peripheral blood cells obtained at the time of bone marrow harvest. In all these patients, we also detected the respective mutation in peripheral blood cells with a close association in VAF between bone marrow–derived and blood-derived cells (*r* = 0.81; *P* = .001).

### Association Between CHIP and Baseline Clinical Characteristics

Table 1 summarizes the baseline characteristics of CHIP and non-CHIP carriers in our cohort of patients with CHF. There were no significant differences except that CHIP carriers were significantly older and more frequently had a history of hypertension than non-CHIP carriers. Except for arterial hypertension, the classic cardiovascular risk factors, such as hyperlipidemia, diabetes mellitus, family history of coronary artery disease, and smoking habits, were not different between CHIP and non-CHIP carriers in our cohort. Likewise, the extent of coronary artery disease was not statistically significantly different between CHIP and non-CHIP patients.

Most importantly, baseline NYHA class, left ventricular ejection fraction, and NT-proBNP serum levels as well as the SHFM score did not differ between the 2 groups (Table 1). Taken together, in patients with CHF owing to ischemic origin, CHIP carriers did not differ from non-CHIP carriers with respect to clinical parameters of heart function and disease stage.

### Prognostic Significance of CHIP in Patients With Heart Failure

**Figure 2.**
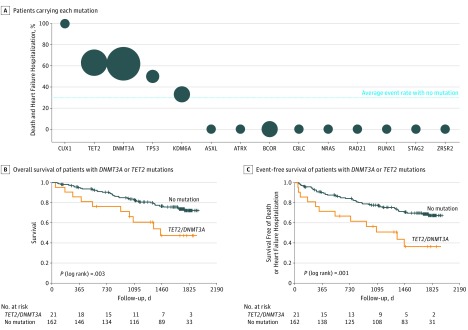
Association Between Mutated Genes and Incidence of Death Combined With Rehospitalization for Heart Failure and Kaplan-Meier Event-Free Survival Curves in Patients With Chronic Heart Failure (CHF) A, Bubble size reflects the sample size of patients carrying each individual mutation, dashed line reflects incidence of death combined with rehospitalization for heart failure for patients who were not carriers of clonal hematopoiesis of indeterminate potential (CHIP). See eTable 1 in the Supplement for gene annotation. B, Overall survival of patients with *DNMT3A* or *TET2* mutations with a variant allele fraction (VAF) of at least 0.02 vs non-CHIP patients. C, Event-free survival free of death or rehospitalization for heart failure with *DNMT3A* or *TET2* mutations with a VAF of at least 0.02 vs non-CHIP patients. *P* values are for log-rank tests.

During a median follow-up of 4.4. years, a total of 53 patients died, and 23 patients required hospitalization for heart failure. Thirty-nine of 162 patients (24%) without CHIP and 14 of 38 (37%) with CHIP died during follow-up. Figure 2A graphically illustrates the relative incidence of death and rehospitalization for heart failure for CHIP carriers with respect to the presence of individual driver gene mutations. The size of the bubbles reflects the sample size of patients carrying each individual mutation. Because most CHIP driver mutations were mutations in *DNMT3A* and *TET2*, as also previously published for atherosclerosis,^4^ and experimental studies demonstrated that those mutations promote cardiac dysfunction in murine models of heart failure,^8,9^ we focused our subsequent analyses on these patients. Patients harboring either *DNMT3A* or *TET2* mutations did not differ from non-CHIP carriers with respect to baseline clinical characteristics, pharmacologic treatment, or device therapy (eTable 3 in the Supplement). However, Kaplan-Meier event-free survival analyses documented a significantly worse long-term clinical outcome for both death (Figure 2B) and death combined with rehospitalization for heart failure (Figure 2C) for patients with CHF of ischemic origin harboring either *DNMT3A* or *TET2* mutations in their bone marrow cells compared with non-CHIP carriers. In patients harboring *DNMT3A* or *TET2* mutations, most deaths (6 of 11) were owing to progression of heart failure and arrhythmia, but there was only 1 death attributed to acute myocardial infarction, and no stroke-related death was observed.

Because age is a major driver of both the presence of CHIP as well as increased risk, stepwise multivariate Cox proportional regression analyses were performed to account for the significant baseline differences. As summarized in Table 2, the presence of somatic mutations within *TET2* or *DNMT3A* remained independently associated with adverse outcome (death or death combined with heart failure hospitalization) in addition to age, whereas hypertension was not independently associated in this multivariable model. Finally, by multivariate Cox proportional regression analysis, including the SHFM score (as tertiles) as well as NT-proBNP serum levels at baseline (stepwise approach using steps of 1000 pg/mL), death as well as the combined event of death and rehospitalization for heart failure also remained independently associated with the presence of *TET2* or *DNMT3A* mutations (death, HR, 3.25; 95% CI, 1.62-6.52; death combined with rehospitalization for heart failure, HR, 3.25; 95% CI, 1.71-6.19), in addition to SHFM score tertiles (death, tertile 2, HR, 6.6; 95% CI, 1.49-29.44 and tertile 3, HR, 26.47; 95% CI, 6.21-112.9) and NT-proBNP serum levels (death, HR, 1.06; 95% CI, 1.00-1.12 per 1000 pg/mL; death combined with rehospitalization for heart failure, HR, 1.11; 95% CI, 1.05-1.17). However, given the limited number of events in the patient cohort as well as the lack of standardized, generally acknowledged stepwise increases in NT-proBNP serum levels or SHFM score values, the HRs should be compared cautiously with the binary yes or no status for the CHIP-associated hazard ratio.

**Table 2.** Multivariable Cox Regression Analyses for Death or Death Combined With Heart Failure Hospitalization

Variable	Hazard Ratio (95% CI)	*P* Value
Death		
Age and *TET2/DNMT3A* mutations		
*TET2* or *DNMT3A *	2.091 (1.043-4.189)	.04
Age	1.037 (1.006-1.069)	.02
Age, hypertension, and *TET2/DNMT3A* mutations		
*TET2* or *DNMT3A*	2.106 (1.047-4.237)	.02
Age	1.042 (1.010-1.076)	.01
Hypertension	0.696 (0.333-1.455)	.34
Death combined with heart failure hospitalization		
Age and *TET2/DNMT3A* mutations		
*TET2* or *DNMT3A*	2.112 (1.115-3.998)	.02
Age	1.040 (1.012-1.069)	.004
Age and *TET2/DNMT3A* mutations		
*TET2* or *DNMT3A*	2.085 (1.100-3.953)	.02
Age	1.041 (1.013-1.071)	.005
Hypertension	0.949 (0.465-1.938)	.89

**Figure 3.**
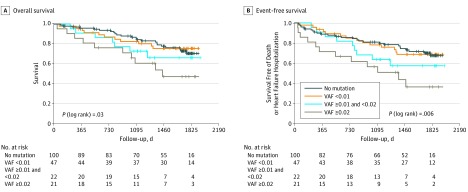
Kaplan-Meier Event-Free Survival Curves for Non–Clonal Hematopoiesis of Indeterminate Potential (CHIP) Carriers and Patients With Clonal Hematopoiesis Caused by *DNMT3A* or *TET2* Mutations at Low Variant Allele Fraction (VAF) A, Overall survival of patients with *DNMT3A* or *TET2* mutations with a VAF greater than 0.005 to 0.01; a VAF of at least 0.01 to 0.02; a VAF of at least 0.02; and non-CHIP patients reveals dose-response of clone size and clinical outcome; B, Event-free survival free of death or rehospitalization for heart failure with *DNMT3A* or *TET2* mutations at various VAFs vs non-CHIP patients. *P* values are for log-rank tests.

To assess a potential association between the extent of clonal expansion caused by *DNMT3A* and *TET2* mutations and clinical outcome, we lowered the threshold of mutation calling to a VAF greater than 0.005. We found 66 additional *DNMT3A* and 53 additional *TET2*-mutated clones with a VAF between 0.005 and less than 0.02 affecting 69 patients. This result confirms a high penetrance of CHIP at low clonal size. To correlate the dosage of clonal expansion with clinical outcome in our patient cohort, we analyzed event-free Kaplan-Meier survival curves for patients with a VAF greater than 0.005 to 0.01; a VAF of at least 0.01 to 0.02; and a VAF of at least 0.02. As illustrated in Figure 3, there was a statistically significant dose-response association between clone size and clinical outcome. These data suggest that there might indeed be a causal association between *DNMT3A* and *TET2* CHIP driver mutations and clinical outcome in patients with CHF.

## Discussion

The results of this study demonstrate that somatic mutations driving clonal hematopoiesis are frequent in patients with chronic ischemic heart failure and the most commonly mutated CHIP driver genes *DNMT3A* and *TET2* are associated with profoundly impaired long-term survival and significantly increased disease progression in patients with CHF of ischemic origin.

While previous studies have documented a significant association of the presence of CHIP with the risk of coronary heart disease in humans,^4^ to our knowledge, this study is the first to show that somatic mutations driving clonal hematopoiesis are associated with profoundly impaired long-term survival and significantly increased disease progression in patients with CHF of ischemic origin. Consistent with previous studies, the most commonly mutated genes in this study were the DNA methyltransferase *DNMT3A* and the DNA demethylase *TET2*. The prevalence of CHIP appears to be significantly higher in the study cohort compared with previously published data in age-matched healthy participants^2^ or patients with coronary artery disease.^4^ Indeed, experimental studies^8,9^ disclosed that both *TET2* and *DNMT3A* loss-of-function in murine hematopoietic stem cells promote cardiac dysfunction in murine models of heart failure.^8,9^ Taken together, these findings suggest that CHIP, at least mediated by mutations in the most prevalent driver genes *DNMT3A* and *TET2*, may contribute to the development of CHF in patients after myocardial infarction.

However, more importantly, this study shows that patients with established CHF harboring mutations in *TET2* or *DNMT3A* demonstrated a profoundly increased mortality during long term follow-up. The increased mortality was mainly driven by a progression of heart failure, but not by the occurrence of acute ischemic events, because only 1 death was owing to a recurring acute myocardial infarction. In addition, impaired clinical outcome associated with *DNMT3A* and *TET2* CHIP driver mutations was independent of the classical prognostic factors SHFM score and NT-proBNP serum levels, which are regarded as the most comprehensive markers of clinical outcome in CHF. Instead, there was a dose-response association between clone size and worse clinical outcome, suggesting that the presence of *DNMT3A* or *TET2* mutations indeed may causally contribute to disease progression in patients with chronic ischemic heart failure. Experimental studies have convincingly demonstrated that mice lacking hematopoietic *TET2* are characterized by augmented IL-1β and inflammasome activation driving atherosclerosis and cardiac dysfunction in murine models of heart failure.^4,7,9^ Moreover, patients with *TET2* clonal hematopoiesis have significantly elevated plasma levels of interleukin-8, the prototypical inflammatory chemokine in humans mediating monocyte adhesion to inflamed endothelium.^4,20^ Likewise, hematopoietic cell deficiency of *DNMT3A* in mice was in 2018 shown to promote inflammation via upregulation of specific cytokines and chemokines in stimulated macrophages.^8^ Because IL-1β neutralizing antibody treatment with canakinumab was shown in 2017 to reduce adverse cardiovascular events in patients with stable coronary heart disease (Cantos Trial),^21^ IL-1β neutralizing antibodies or broader inflammasome inhibitors may provide a targeted therapy for patients with CHF carrying mutations in *TET2* and *DNMT3A*. Thus, although our study cannot provide a definitive causal relationship between the presence of clonal hematopoiesis and the significantly worse prognosis in patients with CHF, it is tempting to speculate that at least CHIP owing to mutations in the most prevalent driver genes *TET2* and *DNMT3A* may be a modifiable risk factor for death in CHF. Additionally, experimental studies reported that *TET2* can be activated by antioxidants such as vitamin C.^22,23^ Therefore, such strategies may also be considered for a targeted therapy augmenting the activity of the remaining wild-type *TET2*.

### Limitations 

Nevertheless, clonal hematopoiesis may also contribute to the progression of heart failure by other mechanisms, including the release of different cardiokines and augmented senescence of BMCs by dysregulation of epigenetic modifiers, stimulation of myofibroblast generation by altered BMCs leading to increased cardiac fibrosis, and altered immunosurveillance. Future studies will have to address the specific mechanisms involved in the progression of heart failure in the presence of clonal hematopoiesis. Likewise, while the presence of CHIP was originally defined as the occurrence of mutations with a VAF of at least 0.02, the results of this study as well as data published in 2018 in healthy patients^24^ indicate that even lower VAFs may confer a survival disadvantage. Further studies are required to potentially identify a specific threshold for variant allele frequency in patients with CHF. However, to make this study comparable with previously published data regarding the role of CHIP in patients with cardiovascular disease, we did limit our conclusions to those patients fulfilling the original classical criteria for CHIP with a VAF of 0.02. The rare occurrence of numerous CHIP driver mutations other than *DNMT3A* and *TET2* in mostly singular patients prevented an assessment of these mutations with respect to clinical outcome. Finally, because the patients studied are not a consecutive series of patients and the sample size is rather moderate, further studies will have to validate our findings in larger cohorts of patients with heart failure. Likewise, the limited number of events precludes the assessment of a potentially added prognostic value of measuring CHIP in addition to NT-proBNP serum levels or the SHFM score in patients with CHF. As such, the calculated respective HRs for the individual prognostic factors should be interpreted with caution.

## Conclusions

In conclusion, our data support the hypothesis that somatic mutations in hematopoietic cells, specifically in the most commonly mutated CHIP driver genes *TET2* and *DNMT3A*, may be significantly associated with the progression and poor prognosis of CHF of ischemic origin. Future studies will have to validate our findings in larger cohorts and to address whether targeting specific inflammatory pathways may be a valuable approach to precision medicine in patients with CHF carrying specific mutations encoding for clonal hematopoiesis.

## Notes

Supplement. **eFigure.** Patient Selection from the Individualized Parent Trials **eTable 1.** List of Genes Analyzed by Custom TruSeq Targeted Sequencing Panel **eTable 2.** CHIP-Associated Somatic Variants Identified in CHF Patients **eTable 3.** Baseline Characteristics of TET2/DNMT3A-mutated and Non-CHIP CHF Patients
