# Supplementary material for: Association of Mutations Contributing to Clonal Hematopoiesis With Prognosis in Chronic Ischemic Heart Failure
Source: JAMA Cardiol. 2018 Dec 19;4(1):32–40. doi: 10.1001/jamacardio.2018.3965 (PMC6439691; doi:10.1001/jamacardio.2018.3965)
Supplement: Supplement. — eFigure. Patient Selection from the Individualized Parent Trials eTable 1. List of Genes Analyzed by Custom TruSeq Targeted Sequencing Panel eTable 2. CHIP-Associated Somatic Variants Identified in CHF Patients eTable 3. Baseline Characteristics of TET2/DNMT3A-mutated and Non-CHIP CHF Patients [file jamacardiol-4-32-s001.pdf]

Supplementary Online Content

Dorsheimer L, Assmus B, Rasper T, et al. Association of mutations contributing to clonal hematopoiesis with prognosis in chronic ischemic heart failure. Published online December 19, 2018. *JAMA Cardiol*. doi:10.1001/jamacardio.2018.3965

eFigure. Patient Selection from the Individualized Parent Trials
eTable 1. List of Genes Analyzed by Custom TruSeq Targeted Sequencing Panel
eTable 2. CHIP0-Associated Somatic Variants Identified in CHF Patients
eTable 3. Baseline Characteristics of *TET2/DNMT3A*-mutated and Non-CHIP CHF Patients

This supplementary material has been provided by the authors to give readers additional information about their work.

© 2018 Dorsheimer L et al. *JAMA Cardiol.*

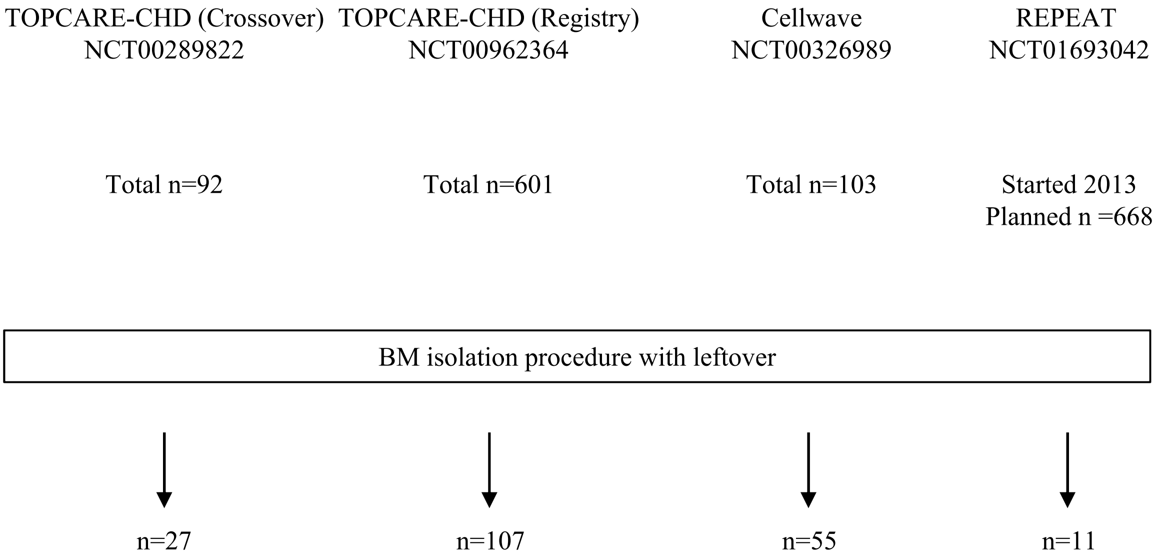

eFigure 1. Patient selection from the individualized parent trials.

© 2018 Dorsheimer L et al. *JAMA Cardiol.*

eTable 1. List of genes analyzed by the custom TruSeq targeted sequencing panel

ABL1	DNMT3A	JAK3	PTPN11
ASXL1	ETV6/TEL	KDM6A	RAD21
ATRX	EZH2	KIT	RUNX1
BCOR	FBXW7	KRAS	SETBP1
BCORL1	FLT3	MLL	SF3B1
BRAF	GATA1	MPL	SMC1A
CALR	GATA2	MYD88	SMC3
CBL	GNAS	NOTCH1	SRSF2
CBLB	GNB1	NPM1	STAG2
CBLC	HRAS	NRAS	TET2
CDKN2A	IDH1	PDGFRA	TP53
CEBPA	IDH2	PHF6	U2AF1
CSF3R	IKZF1	PPM1D	WT1
CUX1	JAK2	PTEN	ZRSR2

© 2018 Dorsheimer L et al. *JAMA Cardiol.*

eTable 2. List of CHIP-associated somatic variants identified in CHF patients

Hugo Symbol	Chr	Position	Variant Classification	Variant Type	Ref. Allele	Alt. Allele	DNA Change	RefSeq	Protein Change	Ref. Reads (CAT A)	Alt. Reads (CAT A)	Ref. Reads (CAT B)	Alt. Reads (CAT B)	VAF
ASXL1	20	31022263	stop_gained	SNP	G	A	c.1748G>A	NM_015338.5	p.W583*	882	64	700	81	0.0677
ASXL1	20	31024387	missense_variant	SNP	T	C	c.3872T>C	NM_015338.5	p.L1291P	121	4	632	4	0.0320
ATRX	X	76872185	missense_variant	SNP	G	A	c.5462C>T	NM_000489.4	p.T1821I	95	2	186	2	0.0206
BCOR	X	39921637	missense_variant	SNP	G	C	c.4183C>G	NM_001123385.1	p.R1395G	448	74	30	7	0.1418
BCOR	X	39930318	missense_variant	SNP	G	A	c.3146C>T	NM_001123385.1	p.A1049V	122	3	162	2	0.0240
BCOR	X	39933926	stop_gained	SNP	G	A	c.673C>T	NM_001123385.1	p.Q225*	627	31	390	29	0.0471
CBLC	19	45297479	missense_variant	SNP	C	T	c.1303C>T	NM_012116.3	p.P435S	34539	1527	123	2	0.0423
CBLC	19	45297479	missense_variant	SNP	C	T	c.1303C>T	NM_012116.3	p.P435S	745	32	100	2	0.0412
CDKN2A	9	21974878	5_prime_UTR_variant	SNP	C	T	c.-52G>A	NM_001195132.1		146	3	295	3	0.0201
CUX1	7	101839979	missense_variant	SNP	C	G	c.1321C>G	NM_001202543.1	p.P441A	121	3	62	2	0.0242
DNMT3A	2	25464430	splice_donor_variant	SNP	C	T	c.2082+1G>A	NM_022552.4		277	16	250	8	0.0546
DNMT3A	2	25457249	missense_variant	SNP	T	C	c.2638A>G	NM_022552.4	p.M880V	388	21	1107	58	0.0513
DNMT3A	2	25457217	frameshift_variant	DEL	GC	G	c.2669delG	NM_022552.4	p.G890fs	316	19	873	42	0.0567
DNMT3A	2	25457243	missense_variant	SNP	G	A	c.2644C>T	NM_022552.4	p.R882C	483	17	1143	83	0.0340
DNMT3A	2	25471084	missense_variant	SNP	G	A	c.677C>T	NM_022552.4	p.A226V	93	2	179	2	0.0211
DNMT3A	2	25471013	missense_variant	SNP	G	A	c.748C>T	NM_022552.4	p.P250S	142	4	249	2	0.0274
DNMT3A	2	25469936	missense_variant	SNP	A	C	c.1106T>G	NM_022552.4	p.I369S	397	91	92	19	0.1865
DNMT3A	2	25458696	splice_acceptor_variant	SNP	T	G	c.2479-2A>C	NM_022552.4		1260	28	572	5	0.0217
DNMT3A	2	25462077	missense_variant	SNP	G	T	c.2330C>A	NM_022552.4	p.P777H	1639	108	587	40	0.0618
DNMT3A	2	25464487	missense_variant	SNP	G	A	c.2026C>T	NM_022552.4	p.R676W	580	13	142	15	0.0219
DNMT3A	2	25469945	missense_variant	SNP	C	T	c.1097G>A	NM_022552.4	p.R366H	191	8	22	3	0.0402
DNMT3A	2	25467156	disruptive_inframe_ deletion	DEL	CTGG GCA	C	c.1713_1718del TGCCCA	NM_022552.4	p.A572_Q5 73del	745	28	414	9	0.0362
DNMT3A	2	25464547	stop_gained; protein_protein_contact	SNP	G	A	c.1966C>T	#	p.Q656*	469	14	131	4	0.0290
DNMT3A	2	25462077	missense_variant	SNP	G	C	c.2330C>G	NM_022552.4	p.P777R	1724	207	644	73	0.1072
KDM6A	X	44732924	missense_variant	SNP	G	C	c.127G>C	NM_001291415.1	p.A43P	194	5	113	3	0.0251
KDM6A	X	44913149	frameshift_variant& missense_variant	complex	AG	T	c.824_825delA GinsT	NM_001291415.1	p.K275fs	231	48	138	44	0.1720

© 2018 Dorsheimer L et al. *JAMA Cardiol.*

Hugo Symbol	Chr	Position	Variant Classification	Variant Type	Ref. Allele	Alt. Allele	DNA Change	RefSeq	Protein Change	Ref. Reads (CAT A)	Alt. Reads (CAT A)	Ref. Reads (CAT B)	Alt. Reads (CAT B)	VAF
KDM6A	X	44941858	frameshift_variant	DEL	CAG	C	c.3340_3341delGA	NM_001291415.1	p.D1114fs	89	2	157	2	0.0220
KDM6A	X	44732828	missense_variant	SNP	G	A	c.31G>A	NM_001291415.1	p.A11T	134	3	180	3	0.0219
NRAS	1	115256464	missense_variant	SNP	C	T	c.247G>A	NM_002524.4	p.A83T	98	2	2032	2	0.0200
RAD21	8	117866614	frameshift_variant& stop_gained	INS	T	TAATC	c.1027_1030dupGATT	NM_006265.2	p.Y344fs	461	77	216	51	0.1431
RUNX1	21	36206842	missense_variant	SNP	G	A	c.670C>T	NM_001754.4	p.R224W	200	5	301	2	0.0244
SF3B1	2	198266834	missense_variant	SNP	T	C	c.2098A>G	NM_012433.3	p.K700E	1310	328	477	114	0.2002
SF3B1	2	198266834	missense_variant	SNP	T	C	c.2098A>G	NM_012433.3	p.K700E	1600	190	595	83	0.1061
SMC1A	X	53432495	missense_variant	SNP	C	T	c.1841G>A	NM_006306.3	p.G614D	179	4	244	2	0.0219
STAG2	X	123227876	missense_variant	SNP	G	A	c.3587G>A	NM_001042749.2	p.G1196D	125	3	68	2	0.0234
TET2	4	106156963	stop_gained	SNP	C	T	c.1864C>T	NM_001127208.2	p.Q622*	986	211	268	68	0.1763
TET2	4	106197104	stop_gained	SNP	C	T	c.5437C>T	NM_001127208.2	p.Q1813*	1325	143	342	45	0.0974
TET2	4	106190804	missense_variant	SNP	G	A	c.4082G>A	NM_001127208.2	p.G1361D	256	6	151	3	0.0229
TET2	4	106193995	stop_gained	SNP	C	G	c.4457C>G	NM_001127208.2	p.S1486*	859	44	142	10	0.0487
TET2	4	106158479	frameshift_variant	DEL	AATATG	A	c.3383_3387delATGAT	NM_001127208	p.Y1128fs	1063	241	311	37	0.1848
TET2	4	106155920	frameshift_variant	DEL	TC	T	c.822delC	NM_001127208.2	p.N275fs	880	52	322	26	0.0558
TET2	4	106164775	stop_gained; protein_protein_contact	SNP	G	T	c.3643G>T; c.3643G>T	NM_001127208.2; NM_001127208.2:4NM6_A:1179_1215	p.E1215*	724	46	111	6	0.0597
TET2	4	106196850	frameshift_variant	INS	A	AGATG	c.5189_5192dupATGG	NM_001127208.2	p.H1732fs	339	17	323	19	0.0478
TET2	4	106164085	splice_donor_variant& intron_variant	SNP	G	C	65256C>G	NM_001127208.2		1126	351	590	182	0.2376
TP53	17	7578442	missense_variant	SNP	T	C	c.488A>G	NM_000546.5	p.Y163C	543	16	395	12	0.0286
TP53	17	7578394	missense_variant	SNP	T	C	c.536A>G	NM_000546.5	p.H179R	380	45	175	25	0.1059
ZRSR2	X	15821875	stop_gained	SNP	A	T	c.268A>T	NM_005089.3	p.K90*	94	2	330	2	0.0208

NM_022552.4;NM_022552.4:4U7T_A:634_656;NM_022552.4:4U7T_A:656_910;NM_022552.4:4U7T_A:656_911;NM_022552.4:4U7T_C:634_656;NM_022552.4:4U7T_C:656_910;NM_022552.4:4U7T_C:656_911

INS insertion
DEL deletion
SNP single nucleotide polymorphism
VAF Variant allele fraction
Ref. reference
Alt. alternate

© 2018 Dorsheimer L et al. *JAMA Cardiol.*

eTable 3. Baseline characteristics of TET2 / DNMT3A-mutated and Non-CHIP CHF patients

	**TET2 or DNMT3A (VAF≥0.02, n=21)**	**Non-CHIP (n=162)**	**P-value (TET2 or DNMT3A versus Non-CHIP)**
Age (years; n=183)	70 ± 8<br>70 (64;75)	62.5 ± 10.8<br>63 (56;71)	**0.004**
Gender (male / female; n; n=183)	17 / 4	137 / 25	0.669
Hypertension (%; n=182)	95	76	**0.048**
Hyperlipidemia (%; n=182)	91	80	0.252
Diabetes mellitus (%; n=182)	33	35	0.852
Current or former smoking (%; n=181)	71	65	0.807
Family history of coronary artery disease (%; n=180)	57	55	0.834
Extent of coronary artery disease: 1/2/3 vessel disease (n; n=180)	1/5/15	45/35/79	0.057
Weight (kg; n=182)	72 ± 12<br>76 (58;82)	82.1 ± 14.1<br>80 (72;90)	0.390
Size (cm; n=182)	170 ± 12<br>174 (158;178)	173.9 ± 8<br>174 (169;179)	0.062
Systolic blood pressure (mmHg; n=170)	121 ± 24<br>120 (101;139)	113.8 ± 22.1<br>111 (100;127)	0.185
Diastolic blood pressure (mmHg; n=168)	60 ± 11<br>58 (50;69)	60.8 ± 10.6<br>60 (53;68)	0.719
Heart rate (min^{-1}; n=158)	64 ± 17<br>61 (59;76)	67 ± 12<br>67 (60;72)	0.235
NYHA class (n=182)	2.4 ± 0.7<br>2 (2;3)	2.3 ± 0.7<br>2 (2;3)	0.810
Time since last myocardial infarction (months; n=170)	125±91<br>138 (36;174)	89±87<br>60 (15;140)	0.094
Seattle Heart Failure Score; (n=166)	0.51 ± 0.97<br>0.363 (-0.117;1.373)	0.4 ± 1.1<br>0.3 (-0.3;0.9)	0.618
Left ventricular ejection fraction (LVEF; %; n=179)	29 ± 9<br>31 (20;35)	30.9 ± 11<br>30 (22;40)	0.519
Severity of mitral regurgitation (grade; n=156)	1.0±0.9<br>1.0 (0;2)	0.8±1<br>1 (0;1)	0.619

© 2018 Dorsheimer L et al. *JAMA Cardiol.*

	TET2 or DNMT3A (VAF≥0.02, n=21)	Non-CHIP (n=162)	P-value (TET2 or DNMT3A versus Non-CHIP)
NT-proBNP serum levels (pg/ml); (n=166)	2129 ± 1668<br>1731 (604;3376)	2032 ± 3872<br>951 (404;1877)	0.912
Creatinine (mg/dl; n=172)	1.2 ± 0.4<br>1.1 (0.8;1.6)	1.3 ± 0.7<br>1.1 (1.0;1.4)	0.610
High sensitivity C-reactive protein levels (mg/dl; n=181)	0.75 ± 1.4<br>0.35 (0.13;0.67)	0.66 ± 1.23<br>0.26 (0.12;0.68)	0.764
High sensitive troponin T levels (pg/ml; n=58)	17 ± 14<br>13 (6;22)	14 ± 9<br>13 (6;20)	0.471
Hemoglobin (g/dl); (n=122)	13 ± 2<br>14 (12;14)	14 ± 2<br>14 (14;15)	0.084
Hematocrit (%); (n=122)	41 ± 4<br>41 (36;44)	42 ± 4<br>42 (39;45)	0.159
Thrombocytes (/µl); (n=122)	204 ± 47<br>197 (163;236)	206 ± 65<br>206 (161;249)	0.878
Leukocytes (/µl); (n=122)	7.4 ± 1.7<br>7.6 (6.2;8.5)	7.8 ± 2.6<br>7.4 (6.3;8.8)	0.618
Previous device therapy<br>- ICD (n=178)<br>- CRT-D (n=183)<br>- CRT-P (n=183)<br>- Pacemaker (n=183)	 9<br>0<br>0<br>6	 62<br>3<br>0<br>20	 0.767<br>0.529<br><br>**0.045**
Renin-Angiotensin-Aldosterone blockade<br>- ACE inhibitor /AT1 blocker (n=181)<br>- Aldosterone blocker (n=123)	 16<br><br>9	 137<br><br>60	 0.261<br><br>0.512
Betablocker (n=181)	20	147	0.588
Statin (n=182)	20	151	0.793
Diuretics (n=182)	20	135	0.167

Continuous variables are shown as mean ± standard deviation; median (interquartile range),

Categorical variables are shown as frequency

© 2018 Dorsheimer L et al. *JAMA Cardiol.*
